# Neurological disability and brain grey matter atrophy in primary progressive multiple sclerosis are determined by microstructural lesional changes, but not by lesion load

**DOI:** 10.1007/s00415-025-13043-x

**Published:** 2025-04-01

**Authors:** Theodoros Ladopoulos, Zainab Abbas, Britta Krieger, Barbara Bellenberg, Jeyanthan Charles James, Jana Bauer, Ralf Gold, Carsten Lukas, Ruth Schneider

**Affiliations:** 1 Department of Neurology, St Josef Hospital, Ruhr University, Gudrunstr. 56, 44791 Bochum, Germany; 2 Institute of Neuroradiology, St Josef Hospital, Ruhr University, Bochum, Germany

**Keywords:** Multiple sclerosis, MRI, Advanced neuroimaging, MDME, Grey matter atrophy, EDSS

## Abstract

**Background:**

Conventional MRI measures, such as the number and volume of MS lesions, are histologically non-specific and cannot sufficiently explain clinical disability or brain atrophy in MS. Nevertheless, demyelinating plaques exhibit distinct histopathological features in relapsing and progressive multiple sclerosis (MS) subtypes. The aim of this study was to assess microstructural characteristics of MS lesions using quantitative MRI and explore their associations with grey matter (GM) atrophy and clinical disability.

**Methods:**

56 control subjects (CS), 121 patients with relapsing–remitting (RRMS), and 38 patients with primary progressive MS (PPMS) underwent 1.5 T MRI scans and clinical examinations. Lesion and brain segmentation based on T1-weighted and FLAIR images were performed using SAMSEG. The MDME sequence and SyMRI software were used to estimate relaxation rates and myelin volume fraction in MS lesions and normal-appearing white matter (NAWM). Associations between quantitative lesional and NAWM MRI parameters with GM atrophy and clinical disability were investigated.

**Results:**

Brain regional volumes and quantitative lesional and NAWM MRI parameters were significantly decreased in patients with PPMS compared to those with RRMS. Quantitative lesional MRI parameters demonstrated statistically significant associations with cortical and deep GM volumes as well as with disability scores in RRMS and especially in PPMS. In contrast to RRMS, lesion volume was not associated with either GM atrophy or clinical disability in the PPMS group.

**Conclusions:**

Quantitative lesional MRI measures, but not lesion load, were strongly associated with clinical disability and GM atrophy in PPMS patients, likely reflecting differences in lesion pathology between MS subtypes.

**Supplementary Information:**

The online version contains supplementary material available at 10.1007/s00415-025-13043-x.

## Introduction

Primary-progressive multiple sclerosis (PPMS) accounts for almost 10% of all multiple sclerosis (MS) cases and is characterized by demyelinating lesions on magnetic resonance imaging (MRI), progressive disability over time, and typical cerebrospinal fluid findings [1, 2]. Although MRI plays a crucial role in diagnosing and monitoring the disease, conventional brain MRI measures (e.g., number and volume of demyelinating lesions) fail to sufficiently explain neurological deficits and brain atrophy. Lesion load shows little-to-no correlation with established disability measures [3–5] or grey matter (GM) atrophy, which is considered one of the most important drivers of disability across all MS subtypes [6, 7]. This mismatch is particularly evident in the progressive phases of MS, whereas in the relapsing form, a more congruent relationship between lesion volume and disability progression has been observed [8].

An important aspect of this discrepancy is that several differences in lesion pathology between progressive and relapsing MS subtypes (e.g., remyelination, axonal degeneration, and destructive smoldering lesions) have been observed, which could have a critical impact on disability and brain atrophy [9]. Unfortunately, tissue hyperintensities in T2-weighted (T2w) or FLAIR (fluid-attenuated inversion recovery) sequences are histologically non-specific and fail to differentiate or quantify pathophysiological processes in MS lesions [10]. Thus, numerous advanced quantitative MRI sequences have been developed over the past 30 years to assess the pathology of demyelinating plaques in detail [11]. Several studies have already demonstrated associations between quantitative lesional MRI measures and physical and cognitive disability as well as with cortical and deep GM atrophy [12]. Tissue relaxometry is one of the most widely used quantitative MRI techniques to evaluate lesion pathology. Relaxation times represent sensitive measures of abnormal tissue integrity and are affected by several pathological processes, such as demyelination, edema, and axonal injury [13]. Several MRI studies have shown significant associations of relaxation times in MS lesions with clinical disability or future disability progression [12, 14]. Nevertheless, the extended examination times and the time-consuming post-processing of most advanced MRI techniques present a barrier to their widespread use, especially in the clinical setting [15].

The advanced multi-delay, multi-echo (MDME) MRI sequence, combined with the SyMRI post-processing software facilitates rapid quantification of relaxation rates (R1, R2) and proton density and estimation of myelin content within almost 7 min [15, 16]. Two histological studies have validated the performance of this sequence for myelin quantification in brains of healthy controls and MS patients [17, 18], and comparative MRI studies have shown good correlation with widely used myelin-sensitive MRI sequences [19, 20]. Interestingly, MDME-derived myelin content in demyelinating plaques has also demonstrated robust associations with standard histological myelin staining [18].

In the present study, we aimed to quantify lesional and NAWM MRI features in PPMS using MDME sequence as a time-efficient quantitative MRI technique. Moreover, we investigated the impact of WM lesion load and quantitative lesional MRI parameters on clinical disability and cerebral cortical (CGM) and deep GM (DGM) atrophy. We hypothesized that microstructural lesional and NAWM characteristics differ between relapsing and primary progressive MS and have a distinct impact on GM atrophy and disability. Therefore, we compared the results from our PPMS cohort with a population of patients with RRMS.

## Methods

### Study population

This retrospective, cross-sectional study incorporated MRI and clinical data from 56 control subjects (CS), 121 RRMS, and 38 PPMS patients. MRI scans were conducted between November 2018 and December as part of routine follow-up examinations. All patients met the 2017 revisions of the McDonald criteria for MS [2]. Exclusion criteria included the presence of any other intracranial pathology unrelated top MS (e.g., brain tumors, cerebral ischemia, and traumatic brain injury), incomplete MRI, and clinical data or insufficient image quality. Demographic and clinical data were obtained from the electronic health records of our hospital. Patients with a clinical relapse or enhancing lesions on MRI scan within the previous 3 months were also excluded. All CS had no history of neurological conditions and revealed no remarkable findings in the neurological examination and MRI scan. The study protocol was approved by the ethics committee of the Medical Faculty at Ruhr-University Bochum (Approval Np. 20–7054-BR).

### MRI acquisition

**Fig. 1:**
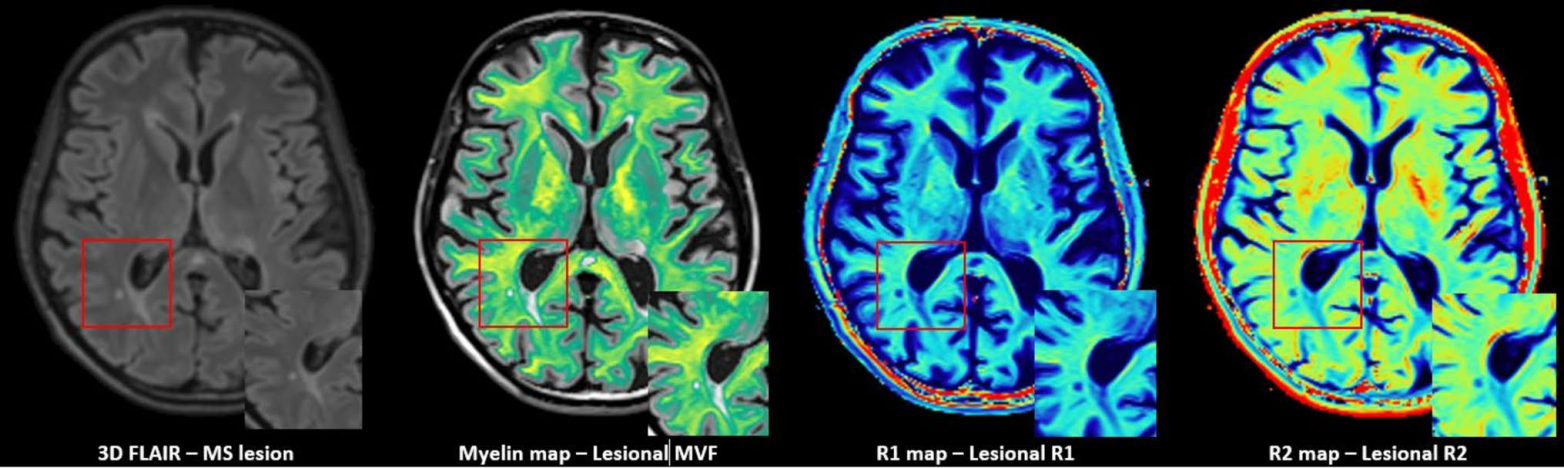
Conventional sagittal 3D FLAIR image and MDME-derived Myelin, R1 and R2 maps. Zoomed image of demyelinating lesions on the lower right corner

Using a 1.5 T MRI scanner (Aera, Siemens Healthineers, Erlangen, Germany) and a 16-channel head/neck matrix coil, the following brain MRI sequences were acquired: (1) MDME sequence (repetition time: 6930 ms, echo time 1: 23 ms, echo time 2: 102 ms, inversion time: 29 ms, acquisition matrix: 256 × 146, voxel size: 1 × 1 × 4 mm^3^), (2) sagittal 3D T1-weighted (T1w) MPRAGE (repetition time: 10 ms, echo time: 4.6 ms, inversion time: 1000 ms, flip angle 8°, acquisition matrix: 240 × 240, voxel size: 1 × 1 × 1 mm^3^, 180 slices), and (3) sagittal 3D FLAIR (repetition time: 5000 ms, echo time: 332 ms, inversion time: 1800 ms, flip angle 120°, number of excitations:1, voxel size 1 × 1 × 1 mm^3^, acquisition matrix: 256 × 230, 160 slices). The MDME sequence was used for quantitative imaging, specifically to quantify myelin volume fractions (MVF) and longitudinal (R1) and transverse (R2) relaxation rates in the whole brain and MS lesions (Fig. 1). This time-efficient advanced MRI technique was described in detail in several previous works by our group and others [15, 16, 18, 20, 21].

### Image analysis

After MDME acquisition, SyMRI Software (Version 12.1.4) was used for automatic estimation of relaxation rates and MVF in the whole brain. In brief, the data from a saturation-recovery and a Carr–Purcell–Meiboom–Gill acquisition are used to fit monoexponential T1 and T2 curves, respectively, and to mathematically obtain proton density (PD) values. Relaxation rates (R1 and R2) were computed by inverting T1 and T2. R1, R2, and PD maps are automatically derived from the SyMRI software and allow the direct extraction of relaxation rate values in whole brain and grey and white matter [15]. MVF quantification is based on a 4-partial-volume-compartment hypothesis which models cellular and extracellular brain microstructure. The relaxation processes in all these compartments contribute to the effective relaxation of every voxel. By incorporating relaxation rates, PD, and magnetization exchange rates of all compartments, it is possible to calculate MVF in each voxel, as previously described [16]. Whole brain MVF was calculated by dividing whole-brain myelin volume with intracranial volume. Based on a single MRI sequence, the SyMRI software generates perfectly registered T1w images, quantitative maps of myelin, R1, R2, and PD and intracranial masks, which we used in the subsequent image analyses.

**Fig. 2:**
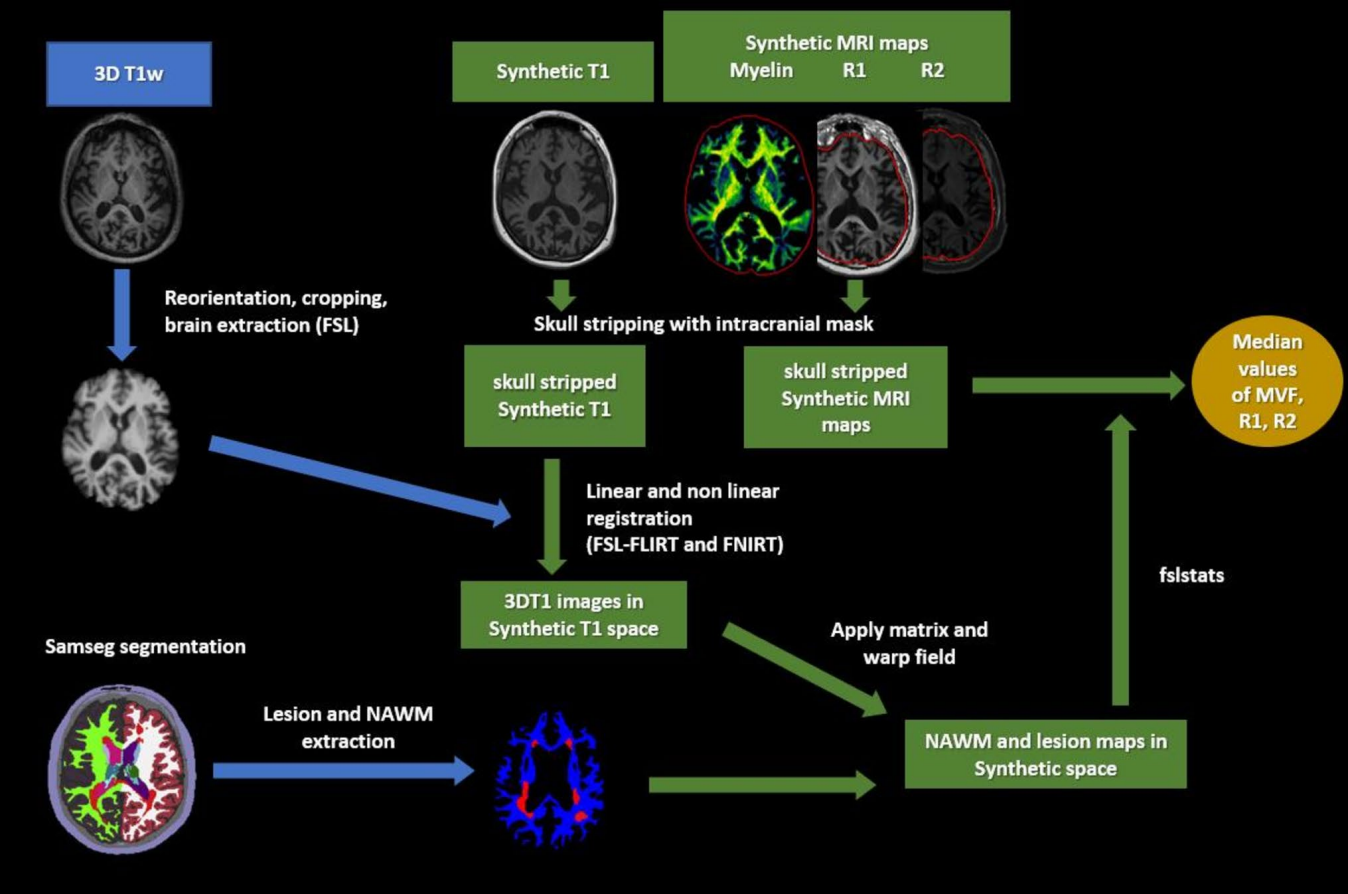
Schematic description of MRI image analysis pipeline

Brain segmentation of the 3D T1w images was performed with FreeSurfer’s Sequence Adaptive Multimodal SEGmentation (SAMSEG) [22]. The 3D FLAIR images were registered to the corresponding T1w image using FreeSurfer’s linear registration program “mri_coreg” and were included in the SAMSEG pipeline to improve the segmentation result and to provide lesion segmentation maps [23]. Analyses were conducted with FreeSurfer’s Version 7.3 [24]. Apart from lesion maps, cerebral CGM, DGM, and NAWM were extracted from the included mesh-based probabilistic atlas as regions of interest (ROIs). Volumetric measures of these ROIs were obtained. Lesion maps were manually corrected using the FSLeyes viewer. The registration of ROIs was reviewed and manually corrected by the corresponding author. The DGM ROI included bilateral putamen, pallidum, caudate nucleus, and thalamus. After binarization of the ROI masks, an FSL (https://fsl.fmrib.ox.ac.uk/fsl/fslwiki) image analysis pipeline was established to provide image registration between the 3D-T1w and the SyMRI-derived images, and estimation of average quantitative MRI parameters within the ROIs (average MVF—aMVF, R1, R2). Reorientation, cropping, and brain extraction were performed for the 3DT1w images using FSL. The SyMRI-derived intracranial mask was used for brain extraction on synthetic T1w images as previously described [21]. The 3D T1w images were linearly and non-linearly (flirt + fnirt) registered to the synthetic T1w images. The resulting matrices and warp fields were used to register each ROI mask from the 3D T1w space to the synthetic T1w space. ROI-based median values and interquartile ranges (IQR) of aMVF, R1 and R2 were estimated using fslstats. Figure 2 presents a schematic description of the MRI image analysis pipeline.

### Statistical analysis

SPSS software (IBM Corp. Released 2016. IBM SPSS Statistics for Windows, Version 26.0. Armonk, NY: IBM Corp.) was used for the statistical analyses. All MRI-based variables were tested for dependency on physiological aging using linear regression models in our CS group. The corresponding inverse transformation for a mean age of 35 years was applied to each MRI variable for all study participants to account for physiological aging effects. In addition, to check for potential confounding effects due to the age difference between the MS groups, we performed a sensitivity analysis on the most important associations and group comparisons reported in the paper by dividing both the PPMS and RRMS groups into subgroups based on the 50th percentile of their respective age distributions. Non-parametric Mann–Whitney and Kruskal–Wallis tests and pairwise post hoc tests for multiple comparisons were performed for group comparisons with the Dunn–Bonferroni correction method. Spearman correlation analyses were used to test for significant associations. ROI volumes were normalized for physiological body-size effects by dividing their values by the corresponding subject´s intracranial volume (ICV) and multiplying the result by the mean ICV of our study population. This method ensures a more accurate and individualized adjustment for brain scaling differences. One PPMS and 19 RRMS patients with extremely low lesion volumes (below the fifth percentile of all MS patients, i.e., < 0.03 ml) were identified as outliers and were excluded from the lesion analysis. Results were considered statistically significant at p < 0.05.

## Results

### Demographic, clinical, and MRI data

The main demographic, clinical, and global MRI features in CS and MS patients are summarized in Table 1. No statistically significant differences regarding the distribution of sexes were observed among groups. PPMS patients had higher median age compared to RRMS patients and CS (p < 0.001). Compared with RRMS, patients with PPMS had significantly longer disease duration and higher EDSS (p < 0.001). In terms of MRI parameters, both, RRMS and PPMS, demonstrated significantly lower whole-brain MVF, normalized WM and GM volumes as well as decreased normalized volumes of cerebral CGM and DGM (p < 0.001) compared to CS. MVF and WM were even significantly lower in PPMS compared to RRMS, while numerical differences in cerebral cortical and deep GM volumes did not reach statistical significance.

**Table 1:** Demographics, clinical, and MRI data

Median (IQR)	CS (n = 56)	RRMS (n = 121)	PPMS (n = 38)	Kruskal–Wallis test
Female (%)	67.9	69.4	57.9	n.s
Age (years)	35 (28–42)	39 (29–49)	58 (54–65)	p < 0.001
Disease duration (years)	–	6 (1–12)	12 (8–20)	p < 0.001
EDSS	–	2 (1.5–3.0)	6.5 (5.0–7.0)	p < 0.001
MVF (%ICV)	10.4 (9.8–11)	9.7 (9–10.3)*	9 (8.3–9.4)^#^,*	p < 0.001
WM volume (ml, age corrected)	449.8 (435.9–463.4)	433.2 (410.9–448.9)*	405.5 (386.9–424.2)^#^,*	p < 0.001
GM volume (ml, age corrected)	654.4 (642.3–670.1)	636.9 (618.3–660.7)*	632 (613.1–646.2)*	p < 0.001
Cerebral CGM volume (ml, age corrected)	515.5 (506.2–533.7)	504.1 (485.5–520.6)*	496.6 (484.4–517.9)*	p < 0.001
DGM volume (ml, age corrected)	35.9 (34.9–37.4)	34 (32.2–35.8)*	33.7 (31.5–34.4)*	p < 0.001
Intracranial volume (ml)	1443 (1395–1559)	1434 (1340–1556)	1504 (1445–1685)	n.s

Values of MVF were derived by SyMRI Software, whereas WM, GM, CGM, and DGM volumes were calculated using SAMSEG

*IQR* interquartile range, *CS* control subjects, *RRMS* relapsing–remitting multiple sclerosis, *PPMS* primary progressive multiple sclerosis, *EDSS* expanded disability status scale, *MVF* myelin volume fraction, *WM* white matter, *GM* grey matter, *CGM* cortical grey matter, *DGM* deep grey matter, *n.s.* not significant

Pairwise group comparison by post hoc Dunn–Bonferroni tests: *significant differences between HC and MS (p < 0.05)

^#^Significant differences between RRMS and PPMS (p < 0.05)

### Quantitative MRI parameters in MS lesions and NAWM

Median values and IQRs of lesion volume and quantitative MRI parameters were compared across groups. The results are summarized in Table 2. Lesion load was significantly higher in PPMS compared to RRMS (p < 0.001). R1 and R2 relaxation rates in MS lesions also exhibited significantly lower values in patients with primary progressive MS (p = 0.001 and p < 0.001, respectively). aMVF values in MS lesions were significantly reduced in PPMS patients compared to RRMS patients (p = 0.012). Statistically significant differences in NAWM aMVF, R1, and R2 values were observed not only between MS patients and CS but also between relapsing and progressive MS subtypes. No associations were found between quantitative MRI parameters in WM and age in the CS group. No significant associations were detected between lesional R1 and aMVF and age of patients with PPMS and RRMS. Lesional R2, however, was significantly associated with age in both MS subtypes (Supplementary Tables 1 and 2).

**Table 2:** Quantitative MRI parameters in MS lesions and NAWM

Median (IQR)	CS (n = 56)	RRMS (n = 102)	PPMS (n = 37)	Kruskal–Wallis test
Lesion volume (ml)	–	3.209 (1.559–7.830)	8.181 (2.826–12.414)	p = 0.001
Lesion aMVF (%)	–	12.1 (11.13–13.13)	10.9 (9.55–12.69)	p = 0.012
Lesion R1 (s^−1^)	–	0.935 (0.875–0.998)	0.867 (0.810–0.912)	p = 0.001
Lesion R2 (s^−1^)	–	8.585 (8.047–9.087)	7.623 (7.228–8.390)	p < 0.001
NAWM aMVF (%)	25.28 (23.99–26.22)	24.21 (22.85–25.11)*	22.88 (21.4–24.52)*,#	p < 0.001
NAWM R1 (s^−1^)	1.445 (1.408–1.482)	1.411 (1.373–1.437)*	1.350 (1.312–1.402)*,^#^	p < 0.001
NAMW R2 (s^−1^)	13.304 (13.09–13.52)	13.011 (12.768–13.216)*	12.722 (12.505–12.982)*,^#^	p < 0.001

*IQR* interquartile range, *CS* control subjects, *RRMS* relapsing–remitting multiple sclerosis, *PPMS* primary progressive multiple sclerosis, *aMVF* average myelin volume fraction, *R1* longitudinal relaxation rate, *R2* transverse relaxation rate, *NAWM* normal-appearing white matter

Pairwise group differences by post hoc tests with Dunn–Bonferroni correction for multiple comparisons: *significant differences between HC and MS subgroup (p < 0.05)

^#^Significant differences between RRMS and PPMS p < 0.05

### Associations between lesional and NAWM quantitative measures and GM volumes

**Fig. 3:**
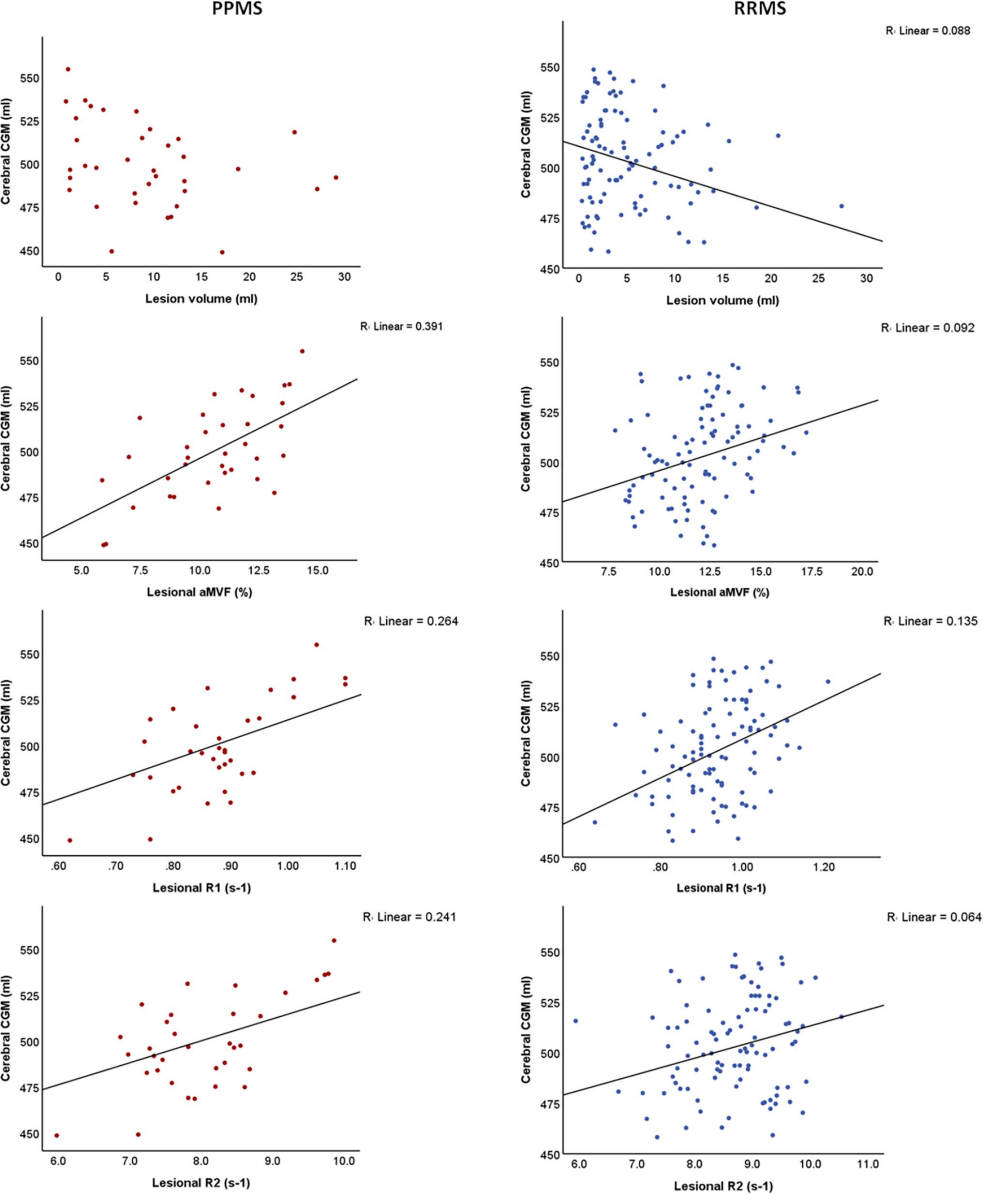
Scatterplots depicting the associations between cerebral CGM and quantitative lesional MRI parameters in patients with PPMS (left) and RRMS (right). *CGM* cortical gray mater, *aMVF* average myelin volume fraction, *RRMS* relapsing–remitting multiple sclerosis, *PPMS* primary progressive multiple sclerosis

Regarding lesion analysis in the PPMS group, we observed significant associations between MDME-derived quantitative metrics and normalized cerebral CGM and DGM volumes (Table 3). Correlation coefficients were highest for aMVF in the WM lesions (0.625 for CGM and 0.593 for DGM). In contrast, no statistically significant correlations were observed between the lesion volume and CGM or DGM (p > 0.05). On the other hand, RRMS patients exhibited significant associations between all MRI parameters, including lesion load and CGM and DGM volumes (Table 3). In RRMS, the lesional R1 also showed the strongest correlation with both cortical GM and DGM. With regard to the DGM volume, the lesion volume even showed the second most significant association (p < 0.003) after the lesional R1 in RRMS. Scatterplots illustrating the associations of quantitative lesional MRI parameters with CGM and DGM are shown in Fig. 3 as well as in Suppl. Fig. 1, respectively.

**Table 3:** Spearman correlation analysis between lesional and NAWM MRI measures and GM volumes; n.s. not significant

Spearman correlations	Normalized age-corrected cerebral CGM volume		Normalized age-corrected DGM volume	
	PPMS (n = 37)	RRMS (n = 102)	PPMS (n = 37)	RRMS (n = 102)
Lesion volume	− 0.302 (n.s.)	− 0.297 (p = 0.002)	− 0.277 (n.s.)	− 0.278 (p = 0.003)
Lesion_aMVF	0.625 (p < 0.001)	0.303 (p = 0.002)	0.594 (p < 0.001)	0.247 (p = 0.013)
Lesion R1	0.514 (p = 0.002)	0.367 (p < 0.001)	0.507 (p = 0.002)	0.288 (p = 0.004)
Lesion R2	0.491 (p = 0.003)	0.253 (p = 0.011)	0.409 (p = 0.015)	0.245 (p = 0.013)
NAWM aMVF	0.392 (p = 0.018)	0.194 (p = 0.04)	0.408 (p = 0.014)	0.219 (p = 0.021)
NAWM R1	0.390 (p = 0.019)	0.237 (p = 0.012)	0.248 (n.s.)	0.242 (p = 0.01)
NAWM R2	0.231 (n.s.)	0.235 (p = 0.012)	0.262 (n.s.)	0.174 (n.s.)

*RRMS* relapsing–remitting multiple sclerosis, *PPMS*: primary progressive multiple sclerosis, *aMVF* average myelin volume fraction, *R1* longitudinal relaxation rate, *R2* transverse relaxation rate, *NAWM* normal-appearing white matter, *CGM* cortical gray matter, *DGM* deep gray matter

The analysis of quantitative MRI parameters in the NAWM in the PPMS group revealed associations between aMVF (p = 0.018) and R1 (p = 0.019) and CGM. Only aMVF in NAWM was associated with normalized DGM volumes (p = 0.014). In the RRMS group, both median relaxation rates and aMVF were significantly associated with CGM volumes. Only aMVF and R1 in NAWM of RRMS patients were associated with DGM volume. In addition, no significant differences were observed in the sensitivity analysis considering different age groups. Results are included in the supplementary material.

### Associations between lesional and NAWM quantitative measure and disability parameters

We observed significant inverse correlations of lesional R1 (p < 0.001) and R2 (p = 0.001) with EDSS in PPMS patients (Table 4). aMVF values also showed an inverse association with EDSS (p = 0.045). No statistically significant association was observed between lesion load and EDSS in the PPMS group. In the RRMS group, moderate but statistically significant correlations were found between lesion load (p = 0.02) and quantitative MRI parameters and EDSS. Apart from NAWM R2 values in the PPMS group, all other quantitative NAWM MRI parameters demonstrated significant inverse associations with EDSS in both groups (Table 4). The sensitivity analysis for different age groups in the two MS subtypes is reported in the supplementary material. Results are in agreement with the main text.

**Table 4:** Spearman correlation analysis between lesional and NAWM MRI measures and EDSS

Spearman correlations	EDSS	
	PPMS (n = 37)	RRMS (n = 102)
Lesion load	0.122 (n.s.)	0.231 (p = 0.02)
Lesion aMVF	– 0.340 (p = 0.045)	– 0.238 (p = 0.017)
Lesion R1	– 0.554 (p < 0.001)	– 0.264 (p = 0.008)
Lesion R2	– 0.518 (p = 0.001)	– 0.284 (p = 0.004)
NAMW MVF	– 0.440 (p = 0.007)	– 0.257 (p = 0.006)
NAWM R1	– 0.376 (p = 0.024)	– 0.261 (p = 0.005)
NAWM R2	– 0.329 (p = 0.05)	– 0.202 (p = 0.033)

*RRMS* relapsing–remitting multiple sclerosis, *PPMS* primary progressive multiple sclerosis, *n.s.* not significant, *aMVF* average myelin volume fraction, *R1* longitudinal relaxation rate, *R2* transverse relaxation rate, *NAWM* normal-appearing white matter, *EDSS* expanded disability status scale

## Discussion

In the present study, we used a fast quantitative MRI sequence to investigate relaxation rates and myelin content in WM lesions and NAWM and their relationship to GM atrophy and disability in relapsing and primary progressive MS. By evaluating quantitative MRI parameters in MS lesions in a large monocentric cohort of patients with MS, who underwent a standardized high-resolution MRI protocol, this study provides valuable insights into lesional and NAWM quantitative MRI features and their impact on clinical disability and GM atrophy in patients with different MS subtypes. We demonstrated that quantitative MRI measures of lesional pathology differed between MS subtypes and were associated with EDSS and cerebral CGM and DGM in relapsing and especially in progressive MS. In contrast, lesion volume was relevant to GM atrophy and disability only in the RRMS group, highlighting differences in pathophysiology between the 2 MS subtypes.

### Lesional and NAWM MRI parameters in relapsing and primary progressive MS

In this study, significant differences between PPMS and RRMS were found in all quantitative lesional and NAWM MRI metrics. The PPMS group demonstrated a higher lesion load compared to RRMS. Similar results have also been reported by multicentric studies in the field using the 2017 revised McDonald criteria for PPMS [6, 25]. Regarding the MDME-derived MRI measures, we observed significant reduction of aMVF and relaxation rates within lesions in PPMS compared to RRMS, suggesting a more destructive lesion pathology in the primary progressive disease course, which is consistent with histopathological studies [26, 27]. Slowly expanding or chronic active lesions are more abundant in progressive MS subtypes than in RRMS and can cause axonal degeneration to a greater extent than classical active lesions [28]. Furthermore, several other pathomechanisms, such as neurodegeneration (including oxidative stress, mitochondrial dysfunction, and excitotoxicity), failure of remyelination due to impaired proliferation and differentiation of oligodendrocytes, and chronic inflammation involving CNS-resident T- and B-cells and microglia, likely contribute to these significant quantitative MRI alterations observed [29].

Progressive MS subtypes are characterized by a compartmentalized inflammatory response in brain, being most extensive in leptomeninges and perivascular spaces and causing diffuse NAWM injury (demyelination, axonal degeneration) [30]. In line with this, we observed a marked decrease of MRI metrics of demyelination (aMVF) and abnormal tissue integrity (R1, R2) in non-lesional WM areas in PPMS patients compared to the CS and RRMS group and to a lesser extent between CS with RRMS patients. We confirmed results from previous quantitative MRI studies using relaxometry and myelin quantification methods, which reported a greater NAWM involvement in progressive than in relapsing MS [31, 32].

Because of the age difference between the MS groups, we investigated associations between quantitative MRI parameters and age. We found no significant association between WM parameters and age in our CS group. Considering that our CS group has a median age of 35, this confirms the previous studies using the MDME sequence that reported an increase in T1 and T2 relaxation times after the age of 60 [33]. Lesional R2 demonstrated a negative association with age in both progressive and relapsing MS. T2 relaxation is heavily influenced by brain iron content [34]. Histopathological studies have shown decreased iron levels in MS lesions with increasing age, which could lead to increased T2 and decreased R2 [35].

### Associations of lesional and NAWM abnormalities with GM atrophy

This study demonstrated differences between relapsing and primary progressive MS regarding the relationships of quantitative lesional MRI parameters and lesion load with GM atrophy. Specifically, we found strong associations between cerebral CGM as well as DGM atrophy and quantitative MRI measures of lesional pathology (aMVF, R1, R2), but not with lesion volume, in patients with PPMS. On the contrary, in RRMS patients, both lesion volume and lesional MRI metrics showed moderate but statistically significant associations with DGM and cerebral CGM atrophy. Lesional aMVF and R1 were—as indicated by correlation coefficients—the most relevant quantitative MRI features for PPMS and RRMS, respectively. Strong associations between MDME-derived MVF and myelin content in healthy WM and demyelination in MS lesions have already been reported, but previous studies failed to demonstrate correlations with disability measures, probably due to small sample sizes [36, 37].

Cerebral CGM and DGM atrophy in MS is thought to result from a combination of pathophysiological mechanisms, including primary disease processes within GM and secondary axonal degeneration due to WM pathology [7]. Large histopathological studies have observed an abundance of destructive smoldering and inactive plaques in PPMS patients [9], whereas remyelinated shadow plaques were fewer compared to RRMS [27]. These findings indicate a more destructive lesion pathology in patients with PPMS, which could increase the likelihood of secondary neurodegeneration in the adjacent or remotely connected brain structures. Since remyelination also differs among individual patients and MS clinical courses and can contribute to axonal preservation, this study provides additional evidence that lesion destructivity could be the actual driver of secondary GM atrophy especially in PPMS and not simply the lesion load [38].

Our findings underpin the importance of quantitative lesional MRI measures in PPMS and the pathogenesis of GM atrophy, since T2w lesion load cannot capture the degree of tissue destruction. The weaker associations in the RRMS group might be explained by more effective remyelination, which potentially protects from axonal death. It is important to note that remyelination can be incomplete and the new myelin structure presents several different features (thinner myelin sheaths, short internodes) compared to normal neurons [39]. This could lead to alterations in MVF and relaxation rates and some incoherence between abnormalities of quantitative MRI metrics and GM atrophy. This likely explains the higher correlation coefficients for lesional R1 (as a marker for axonal neurodegeneration) in the RRMS group compared to aMVF and indirectly indicates a more effective remyelination process in relapsing MS. Conversely, lesional aMVF demonstrated the strongest correlations with GM in the PPMS group pointing to more destructive non-repairable demyelination processes which lead to neurodegeneration and GM atrophy. Intriguingly, quantitative MRI measures of NAWM appear subordinate compared to MS lesions in both MS subtypes, underscoring the importance of demyelination in plaques as pathophysiologic hallmark of the disease.

### Lesional and NAWM correlates of clinical disability

We observed statistically significant associations between disease disability, as measured by EDSS, and quantitative lesional MRI parameters. Again, there was no association between lesion load and EDSS in the PPMS group but significant correlations between EDSS and lesional and NAWM quantitative MRI metrics. R1 and R2 median values in the PPMS lesions demonstrated slightly stronger associations with clinical disability compared with lesional myelin. Unlike MVF, alterations in relaxation rates encompass a wider spectrum of WM pathologies, including axonal degeneration, which leads to neuronal death and probably plays a more crucial role in disability accumulation [13, 18]. In contrast to the PPMS group, the RRMS group consistently demonstrated significant associations between lesion load, MRI metrics, and EDSS suggesting the aforementioned different pathophysiological background.

### Limitations

We acknowledge that the present study has some limitations. First, a key demographic feature of our study population is that the median age between the 2 MS groups is significantly different. PPMS typically manifests later and has a more devastating course—due to the lack of effective disease-modifying treatments—than RRMS. Considering this, it is difficult to match the 2 MS subtypes for age and disease disability. Furthermore, matching the 2 MS groups could mask disease-specific pathophysiologic effects. To address potential confounding age effects, we conducted a sensitivity analysis on the most important correlation analyses and group comparisons. The results were consistent with those presented in the main text, further supporting the generalizability of our findings across different age groups. Apart from R2, no significant association between quantitative MRI parameters and age of the participants was observed in our analysis. Second, we evaluated lesional and NAWM MRI measures as markers of microstructural pathology in a cross-sectional manner. Given the exploratory nature of our study, future longitudinal studies are essential to investigate the dynamics of lesional and NAWM pathology, as well as their relationship with GM atrophy and clinical disability over time. Furthermore, other advanced statistical techniques (e.g., structural equation modeling) can help explore causal relationships and directionality of the associations observed. Third, our study did not include an evaluation of cortical lesions by virtue of lacking suitable MRI sequences at an appropriate magnetic field strength (e.g., FLAIR, double inversion recovery, magnetization-prepared 2 rapid gradient echo, and phase-sensitive inversion recovery sequences at 3 T or 7 T MRI scanners). As several studies have indicated, cortical lesions can lead to cortical atrophy along with WM lesions and NAWM pathology [40]. Future studies should use higher field MRI sequences and incorporate cortical lesion number and lesion volume to clarify their contribution to clinical disability and GM atrophy. Fourth, the discrepancy between our results and past studies may be attributed to the revised 2017 McDonald criteria and the methodological heterogeneities among advanced MRI protocols. Fifth, we acknowledge that our study excluded patients with active disease (clinical relapses or new enhancing lesions), and as such, our results may not be applicable to this patient population. Furthermore, although we included the evaluation of NAWM pathology, our study primarily focuses on MS lesion alterations and their relationship with disability and GM atrophy. Other MRI techniques, such as diffusion tensor imaging (DTI), are presumably better suited for evaluating NAWM pathology due to their greater sensitivity and topological accuracy. Finally, the size of the PPMS group of patients could be a limitation, although it is larger than in other advanced MRI studies that investigated similar topics [41, 42]. Recruiting a sufficient number of PPMS patients in a monocentric setting is challenging, especially when they need to be scanned using a standardized protocol on the same MRI scanner. Nevertheless, the relatively small sample size particularly in the PPMS group may limit the generalizability of our results. Conducting a multicentric study with standardization of the MDME sequence across different MRI scanners could help mitigate this limitation and address the sample size issue.

## Conclusion

This work provides evidence that in patients with PPMS lesion, destructivity characterized by quantitative MRI measures is more relevant to subsequent GM atrophy and disability than lesion load. Besides demonstrating critical differences between MS subtypes, MRI measures of lesion pathology, but not lesion load, were associated with clinical disability and GM atrophy in PPMS patients, probably reflecting the lack of effective remyelination and severe lesion pathology in the primary progressive disease type. Considering the time efficiency and accuracy of the MDME sequence, which provides a comprehensive analysis of brain tissue properties and has shown promising associations with clinical measures, these results have potential clinical utility. Due to the short scanning time and the high number of diagnostic quantitative measures to be collected, the sequence is particularly interesting for pediatric populations [43]. However, further longitudinal studies are needed to validate the impact of these metrics on disability progression and elucidate lesion dynamics across MS subtypes. Despite current limitations, its applicability at different MRI field strengths and its short scanning time make it a promising tool for assessing MS-related tissue changes in clinical settings.

## Supplementary Information

Below is the link to the electronic supplementary material.

Supplementary file1 (DOCX 205 KB)

## Data Availability

The datasets for this article are not publicly available due to concerns regarding participant/patient anonymity.

## Abbreviations

MS: Multiple sclerosis
PPMS: Primary progressive multiple sclerosis
RRMS: Relapsing–remitting multiple sclerosis
MRI: Magnetic resonance imaging
GM: Grey matter
CGM: Cortical grey matter
DGM: Deep grey matter
T2w: T2-weighted
FLAIR: Fluid attenuated inversion recovery
QRAPMASTER: Quantification of relaxation times and proton density by multi-echo acquisition of a saturation recovery using turbo spin-echo readout
CS: Control subjects
EDSS: Expanded Disability Status Scale
T1w: T1-weighted
MVF: Myelin volume fraction
R1: Longitudinal relaxation rate
R2: Transversal relaxation rate
PD: Proton density
SAMSEG: Sequence adaptive multimodal segmentation
NAWM: Normal-appearing white matter
ROI: Region of interest
aMVF: Average MVF
IQR: Interquartile range
