# Supplementary material for: Neurological disability and brain grey matter atrophy in primary progressive multiple sclerosis are determined by microstructural lesional changes, but not by lesion load
Source: J Neurol. 2025 Apr 1;272(4):302. doi: 10.1007/s00415-025-13043-x (PMC11961454; doi:10.1007/s00415-025-13043-x)
Supplement: Supplementary file 1 — Supplementary file1 (DOCX 205 KB) [file 415_2025_13043_MOESM1_ESM.docx]

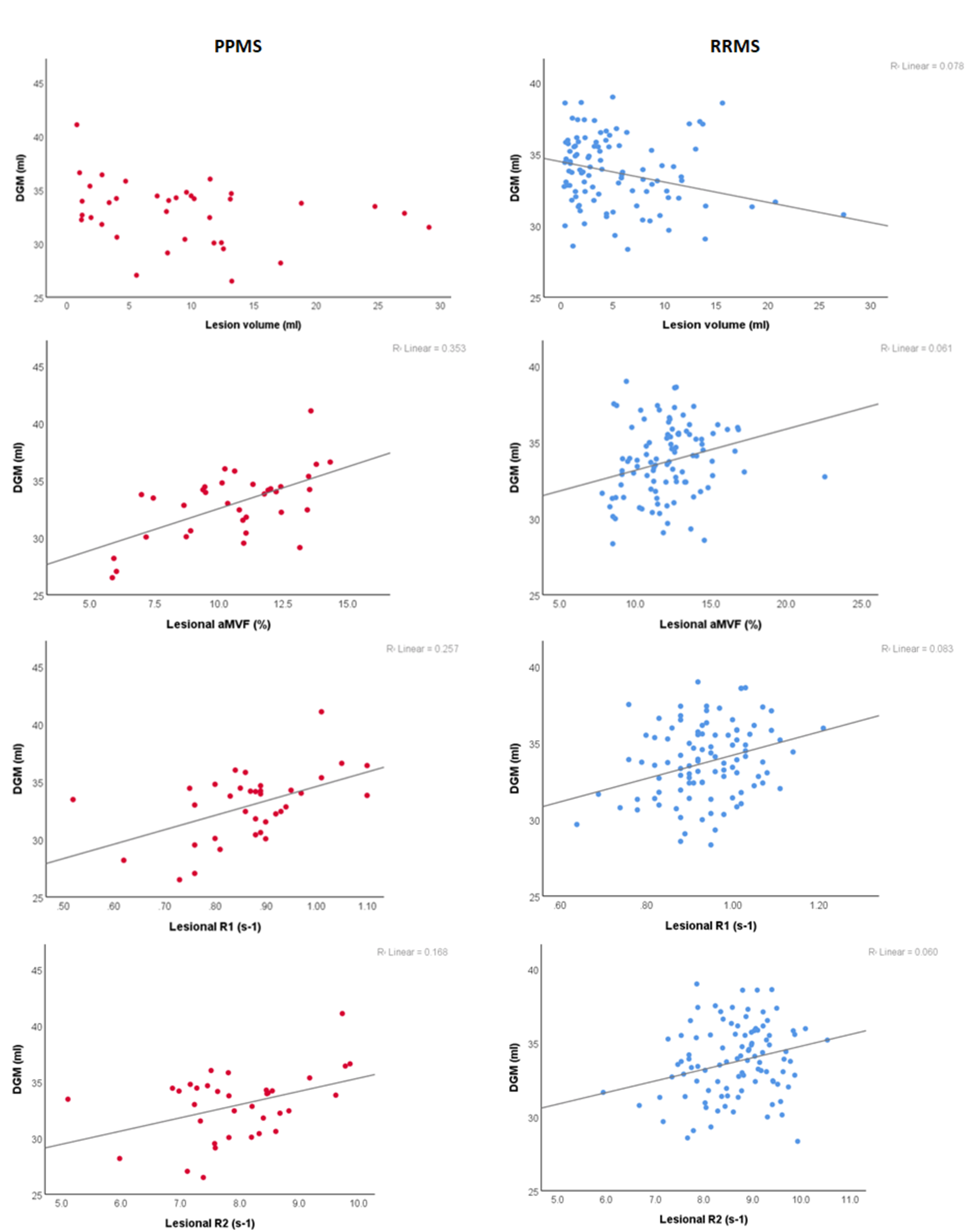


**Suppl. Figure 1.** Scatterplots depicting the associations between cerebral DGM and quantitative lesional MRI parameters in patients with PPMS (left) and RRMS (right). Abbreviations: DGM deep gray mater, aMVF: average myelin volume fraction, RRMS: relapsing remitting multiple sclerosis, PPMS: primary progressive multiple sclerosis

|  | Age |
| --- | --- |
| WM aMVF | n.s. (p>0.05) |
| WM R1 | n.s. (p>0.05) |
| WM R2 | n.s. (p>0.05) |

**Supplementary Table 1**. Spearman correlation analysis in the CS group between quantitative MRI parameters in WM and age of the participants.

|  | PPMS | RRMS |
| --- | --- | --- |
| Lesion aMVF | n.s. (p>0.05) | n.s. (p>0.05) |
| Lesion R1 | n.s. (p>0.05) | n.s. (p>0.05) |
| Lesion R2 | -0.478 (p= 0.004) | -0.264 (p=0.007) |

**Supplementary Table 2.** Spearman correlation analysis in the PPMS and RRMS group between quantitative MRI parameters in MS and age of the participants.

**Sensitivity analysis for different age groups**

**Group A Age (years):** HC = 29 (24-32) , PPMS = 52 (45 -56), RRMS= 31 (28-37)

**Group B Age (years):** HC= 53 (40-52), PPMS= 61 (59-65), RRMS= 50 (45-57)

**Group A (Age 0^th^ – 50^th^ percentile, IQR)**

| **Spearman correlations** | **Normalized age corrected cerebral CGM volume** | | **Normalized age corrected DGM volume** | |
| --- | --- | --- | --- | --- |
|  | PPMS (n=18) | RRMS (n=52) | PPMS (n=37) | RRMS (n=62) |
| **Lesion volume** | -0.288 (n.s.) | -0.348 (p=0.011) | -0.291(n.s.) | -0.203(p=0.035) |
| **Lesion_aMVF** | 0.662(p=0.004) | 0.213 (n.s.) | 0.725 (p=0.001) | 0.252(p=0.041) |
| **Lesion R1** | 0.737 (p=0.001) | 0.371(p=0.007) | 0.804 (p=0.002) | 0.269 (p=0.033) |
| **Lesion R2** | 0.814 (p<0.001) | 0.287 (p=0.039) | 0.873 (p<0.001) | 0.191 (n.s.) |

**Supplementary Table 3.** Spearman correlation analysis between lesional MRI measures and GM volumes, n.s. not significant. RRMS relapsing remitting multiple sclerosis, PPMS: primary progressive multiple sclerosis, aMVF average myelin volume fraction, R1 longitudinal relaxation rate, R2 transverse relaxation rate, CGM cortical gray matter, DGM deep gray matter.

| **Spearman correlations** | **EDSS** | |
| --- | --- | --- |
|  | PPMS (n=18) | RRMS (n=52) |
| **Lesion load** | 0.263 (n.s.) | 0.245 (p=0.041) |
| **Lesion aMVF** | -0.339 (n.s.) | -0.259 (p=0.035) |
| **Lesion R1** | -0.519 (p=0.03) | -0.281 (p=0.02) |
| **Lesion R2** | -0.418 (p=0.04) | -0.173 (n.s.) |

**Supplementary Table 4.** Spearman correlation analysis between lesional MRI measures and EDSS. RRMS relapsing remitting multiple sclerosis, PPMS: primary progressive multiple sclerosis, n.s. not significant, aMVF average myelin volume fraction, R1 longitudinal relaxation rate, R2 transverse relaxation rate, EDSS expanded disability status scale

**Group B (Age 51^th^ – 100^th^ percentile, IQR)**

| **Spearman correlations** | **Normalized age corrected cerebral CGM volume** | | **Normalized age corrected DGM volume** | |
| --- | --- | --- | --- | --- |
|  | PPMS (n=19) | RRMS (n=50) | PPMS (n=37) | RRMS (n=62) |
| **Lesion volume** | -0.2130(n.s.) | -0.345 (p=0.014) | -0.291(n.s.) | -0.227(p=0.04) |
| **Lesion_aMVF** | 0.609(p=0.008) | 0.401 (p=0.004) | 0.661 (p=0.001) | 0.212 (n.s.) |
| **Lesion R1** | 0.591 (p=0.03) | 0.461(p=0.001) | 0.479 (p=0.04) | 0.367 (p=0.01) |
| **Lesion R2** | 0.576 (p=0.004) | 0.356 (p=0.012) | 0.334 (n.s.) | 0.393 (p=0.005) |

**Supplementary Table 5**. Spearman correlation analysis between lesional MRI measures and GM volumes, n.s. not significant. RRMS relapsing remitting multiple sclerosis, PPMS: primary progressive multiple sclerosis, aMVF average myelin volume fraction, R1 longitudinal relaxation rate, R2 transverse relaxation rate, CGM cortical gray matter, DGM deep gray matter.

| **Spearman correlations** | **EDSS** | |
| --- | --- | --- |
|  | PPMS (n=19) | RRMS (n=50) |
| **Lesion load** | -0.07 (n.s.) | 0.251 (p=0.041) |
| **Lesion aMVF** | -0.103 (n.s.) | -0.309 (p=0.031) |
| **Lesion R1** | -0.485 (p=0.041) | -0.382 (p=0.007) |
| **Lesion R2** | -0.519 (p=0.027) | -0.441(p=0.002) |

**Supplementary Table 6.** Spearman correlation analysis between lesional MRI measures and EDSS. RRMS relapsing remitting multiple sclerosis, PPMS: primary progressive multiple sclerosis, n.s. not significant, aMVF average myelin volume fraction, R1 longitudinal relaxation rate, R2 transverse relaxation rate, EDSS expanded disability status scale

**Group A (Age 0^th^ – 50^th^ percentile, IQR)**

| **Median (IQR)** | **CS (n=28)** | **RRMS (n=52)** | **PPMS (n=18)** | **Kruskal Wallis** |
| --- | --- | --- | --- | --- |
| **Lesion volume (ml)** | - | 2.63 (0.7-5.64) | 3.69 (1.86 – 11.47) | P=0.03 |
| **Lesion aMVF (%)** | - | 11.8 (10.4-12.8) | 10.9 (9.5 -13.5) | P=0.002 |
| **Lesion R1 (s^-1^)** | - | 0.94 (0.89-1.01) | 0.89(0.86 – 1.01) | P=0.01 |
| **Lesion R2 (s^-1^)** | - | 8.9 (8.1-9.4) | 8.4 (7.91-9.17) | n.s. |
| **NAWM aMVF (%)** | 25.8 (24.0-26.7) | 24.5 (22.8-25.5) * | 24.1 (23.1-25.3) *, # | P<0.001 |
| **NAWM R1 (s^-1^)** | 1.45 (1.41-1.49) | 1.42 (1.38-1.44) * | 1.39 (1.37-1.44) * | P=0.04 |
| **NAMW R2 (s^-1^)** | 13.4 (13.0-.13.6) | 13.2 (12.8-.13.4) * | 12.9(12.7-.13.3) * | P=0.03 |

**Supplementary Table 7.** Quantitative MRI parameters in MS lesions and NAWM. Pairwise group differences by post-hoc tests with Dunn–Bonferroni correction for multiple comparisons: *significant differences between HC and MS subgroup (p<0.05), #significant differences between RRMS and PPMS p<0.05. Abbreviations: IQR interquartile range, CS control subjects, RRMS relapsing remitting multiple sclerosis, PPMS primary progressive multiple scleoris, aMVF average myelin volume fraction, R1 longitudinal relaxation rate, R2 transverse relaxation rate, NAWM normal appearing white matter

**Group B (Age 51^th^ – 100^th^ percentile, IQR)**

| **Median (IQR)** | **CS (n=28)** | **RRMS (n=50)** | **PPMS (n=19)** | **Kruskal Wallis** |
| --- | --- | --- | --- | --- |
| **Lesion volume (ml)** | - | 4.09 (1.43-8.78) | 9.98 (7.25-17.20) | P=0.002 |
| **Lesion aMVF (%)** | - | 12.3 (11.1-13.8 | 10.2 (8.6-11.3) | P=0.01 |
| **Lesion R1 (s^-1^)** | - | 0.93 (0.86-1.03) | 0.85 (0.76-0.89) | P=0.004 |
| **Lesion R2 (s^-1^)** | - | 8.4 (7.7-9.1) | 7.4 (7.1-7.9) | P=0.001 |
| **NAWM aMVF (%)** | 25.3 (23.7-25.9) | 23.9 (22.8-24.9) * | 21.7 (20.9-22.7) *, # | P<0.001 |
| **NAWM R1 (s^-1^)** | 1.44 (1.39-1.46) | 1.40 (1.36-1.42) * | 1.33 (1.28-1.35) *, # | P<0.001 |
| **NAMW R2 (s^-1^)** | 13.3 (13.1-13.4) | 13.0 (12.7-13.2) * | 12.6 (12.3-12.8) *, # | P=0.003 |

**Supplementary Table 8.** Quantitative MRI parameters in MS lesions and NAWM. Pairwise group differences by post-hoc tests with Dunn–Bonferroni correction for multiple comparisons: *significant differences between HC and MS subgroup (p<0.05), #significant differences between RRMS and PPMS p<0.05. Abbreviations: IQR interquartile range, CS control subjects, RRMS relapsing remitting multiple sclerosis, PPMS primary progressive multiple scleoris, aMVF average myelin volume fraction, R1 longitudinal relaxation rate, R2 transverse relaxation rate, NAWM normal appearing white matter
